# The Accuracy of 3D‐Printed Fixed Dental Restorations

**DOI:** 10.1111/jerd.13365

**Published:** 2024-12-08

**Authors:** Amirah Alammar, Wael Att, Florian Beuer

**Affiliations:** ^1^ Department of Prosthodontics University Hospital Berlin Charité Berlin Germany; ^2^ Founder and Director The Face Dental Group Boston Massachusetts USA

**Keywords:** 3D‐printing, accuracy, CAD/CAM, DLP, FDPs, polymer‐based materials, SLA

## Abstract

**Objective:**

The aim of this in vitro study was to evaluate the accuracy of resin‐based fixed dental restorations, namely veneers, single crowns, and four‐unit fixed partial dental prosthesis (FPDs), using two different 3D printing technologies and polymer‐based materials.

**Materials and Methods:**

A standard maxillary polyurethane jaw model containing prepared teeth was scanned using an intraoral scanner. The generated STL data were used to design the restorations virtually using CAD software. Two 3D printers were utilized for the provisional digital light processing and stereolithography for the castable resin patterns. Each printer produced 10 specimens of each type of restoration, for a total of 80 restorations. The 3D‐printed restorations were then 3D scanned using the same intraoral scanner and evaluated for external and internal dimensional accuracy in terms of trueness and precision. A one‐way ANOVA and two‐sample T‐test were implemented to compute the precision (variability between groups) and trueness (with the designed CAD model). A level of statistical significance of *p*‐value < 0.05 was set.

**Results:**

Statistical differences in the external dimensional analysis of the incisors, molars, and four‐unit FPD with *p*‐values < 0.001, 0.002, and 0.004, respectively. For the internal dimensional analysis, the overall mean values of trueness ranged between 17 and 52 μm, and the variability was significant.

**Conclusion:**

The external and internal dimensional accuracy values of the 3D‐printed fixed dental restorations in this in vitro study in terms of trueness can be clinically accepted after chairside modifications. However, significant variability between the 3D‐printed restorations was observed. Further investigations are needed to improve the accuracy of the 3D‐printed fixed dental restorations.

**Clinical Significance:**

In terms of clinical applications, 3D‐printed fixed dental restorations produced by both 3D‐printing technologies and polymer‐based materials achieved acceptable levels of trueness, although some variability was observed. Significant deviations from the CAD model may require further chairside adjustments. Future integration of AI with 3D‐printing may further improve the accuracy and efficiency of fixed dental restoration production.

## 1 Introduction

Digital dental manufacturing technology has advanced tremendously in recent years. Dental restorations produced with computer assistance are now common in daily dental practice. Increased patient satisfaction [1], infection prevention [2], reduced office chair time [3], and decreased cost and material expenses [4], as well as the higher accuracy of treatment outcomes are the main features promoted as the advantages of the use of computer‐aided restorations [5].

Computer‐aided or computer‐assisted design/computer‐aided manufacturing (CAD/CAM) is a technology used to produce different types of prostheses, including crowns, veneers, inlays, onlays, fixed dental prostheses (FDPs), removable dental prostheses (RPDs), dental implant prostheses, and orthodontic and other devices [6]. All CAD/CAM technologies have digitalization tools that transform geometries into digital data processed by a computer, a software program that designs virtual restorations, and a production technology that fabricates the designed restoration [7]. CAD/CAM technologies are divided into subtractive, additive, and hybrid [8]. Subtractive technology uses a computer numerically controlled (CNC) milling machine that subtracts the material from solid prefabricated blocks by using sharp cutting tools [9]. Despite the fact that this technology fabricates precise restorations, 90% of the prefabricated block is removed and the wasted material cannot be reused [10], which can also be unfavorable for the environment [11]. Another limitation of this technology is the inability to fabricate complex geometrical restorations due to the restricted accessibility of the burs during the subtraction process [12]. Occlusal anatomy and fine surface details of the restorations are difficult to produce [13].

Additive manufacturing technology or 3D printing is the inverse of subtractive technology. It is defined as a manufacturing process that builds three‐dimensional structures by depositing layers of material on top of each other until the final structure is achieved. 3D printing can produce objects made of single or multiple materials without being limited by undercuts or complexity [14]. Hybrid manufacturing technology is a combination of both subtractive and additive [15, 16]. Restorations fabricated through this technology combine the efficiency of the additively fabricated geometrical complex restorations and the precision of the subtractive [8].

3D printing was first implemented in medicine and dentistry, with advances leading to the production of parts with internal features, growth factors, proteins, and cell patterning [17]. Other names of this technology include additive fabrication, additive processes, direct digital manufacturing, rapid prototyping, rapid manufacturing, layer manufacturing, and solid freedom fabrication [18]. The International Organization of Standardization (ISO) provided an overview of the 3D‐printing process used in dentistry and divided it into seven main categories: vat photopolymerization, material extrusion, powder‐based fusion, material jetting, binder jetting, direct energy deposition, and sheet lamination [19]. Each of these categories has its own set of dental applications, materials, and manufacturing protocols. The American Society for Testing and Materials (ASTM) Committee also categorized 3D‐printing technologies based on machine processes [19]. Furthermore, 3D‐printing technologies can also be categorized according to the material state: (1) liquid, (2) filament/paste, (3) powder, and (4) solid sheet [20].

The main idea of this technology is that after virtualizing the restoration using 3D CAD software and exporting it to the 3D printer, the exported file is sliced into a stack of two‐dimensional planar layers. The selected material is then laid over the working surface in thin layers and every new layer is formed and bonded on top of the previous layer until the restoration is formed. 3D‐printed layers are controlled by an energy source and it depends on the selected 3D‐printed process [19].

Various 3D‐printing technologies with different materials, such as polymers, metal alloys, and ceramics, can be applied in dentistry. Polymer‐based 3D‐printed prosthetic applications include diagnostic and definitive casts, interim restorations, castable patterns, custom trays, silicone indices, complete dentures, deprogrammers, and occlusal devices. Metal‐based applications include frameworks for removable prostheses, tooth‐and implant‐supported dental restorations, and splinting frameworks for complete arch impression techniques. Ceramic‐based applications include lithium disilicate and zirconia tooth‐supported restorations [21, 22, 23, 24].

For prosthetic restoration 3D‐printing, several steps are involved. Using CAD software, the external or internal geometry of the restoration is designed in an unordered set of planar triangles. The CAD software generates a standard tessellation language (STL) file. The STL file consists of three vertices (namely, X, Y, and Z coordinates) and an index that describes the orientation of the restoration. After this step, the designed restoration is mathematically sliced into layers with a plane parallel to the horizontal plane and sent to a 3D printer. The 3D‐printing process starts by fabricating support structures and then each layer is printed according to the given layer thickness, and then bonded to the preceding layer until the restoration is completed [25]. 3D‐printed restorations often require additional hardening, cleaning, and finishing procedures, known as a post‐processing step. Restorations in this step are relatively unstable and have supporting structures that must be removed [26].

Despite continual advancements in 3D printing technology, determining whether it can compete with other digital dental manufacturing technologies remains difficult. SLA and DLP 3D printing technologies are the most widely used technologies in fabricating FDPs [27]. Both of them are categorized as polymer‐based, but the main difference between them is the technique of building the layers [28].

SLA was the first commercially available system introduced to the market as it offers the highest levels of accuracy, a smooth surface finish, and good chemical bonding between layers [29, 30]. This process uses a liquid resin vat, a build platform, and an ultraviolet (UV) laser in a “top‐down” approach [29]. The UV laser begins by tracing the outline of the first layer onto the resin's surface point at point [31]. After the first layer is fully cured based on the CAD model, a resin‐coated blade sweeps across the cross section of the object, adding a new layer of resin. The platform then lowers slightly in the Z‐direction, allowing the next layer to be cured [32]. This process repeats until the entire object is completely fabricated. The main drawbacks are limited longevity and low flexural strength [33]. DLP is faster than SLA in the fabrication and wastes less material, which reduces the cost [21]. It uses a digital projector as a light source instead of a laser and cures the entire layer at the same time [34]. The projector is based on a micro‐electro‐mechanical system (MEMS) with a digital mirror device (DMD). During printing, the build platform (x‐y axis) is lowered into the resin vat by the thickness of one layer [34]. Light is then reflected onto the resin surface by the DMD, solidifying it to form the layer according to the CAD design [34]. The DMD's micro‐mirrors individually control the direction of the light. After each layer is formed, the build platform is raised (z‐axis) by one layer's thickness, and this process is repeated until the object is fabricated [35, 36].

In vitro studies gave promising results regarding the accuracy of some types of resin‐based 3D‐printed dental restorations [37, 38], while other studies found that they were inaccurate to be clinically accepted and further investigations were essential and found different behavior of polymer‐based materials [39, 40, 41].

Therefore, the aim of this in vitro study is to evaluate the accuracy of 3D‐printed fixed dental restorations; namely, veneers, incisors, molars, and four‐unit fixed partial dentures (FPDs), using two different 3D‐printing technologies and polymer‐based materials. The working hypothesis of this study is that both SLA and DLP polymer‐based 3D‐printed FDPs will provide acceptable accuracy values in terms of trueness and precision when compared to the original STL data.

## 2 Material and Methods

### 2.1 3D‐Printing Process

**FIGURE 1:**
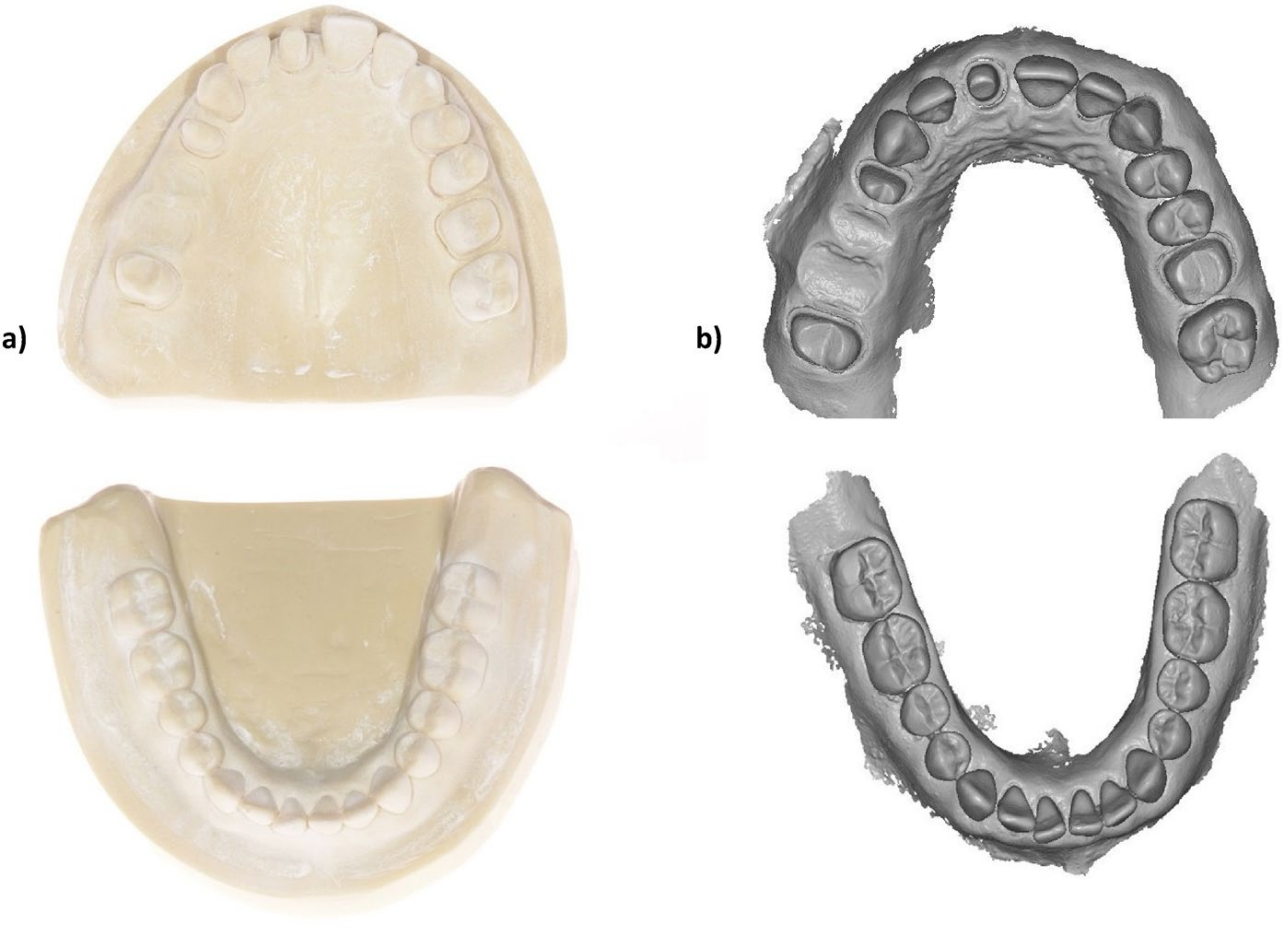
(a) Polyurethane reference jaw models; (b) volumetric information obtained by the True Definition IOS.

For FDPs 3D‐printing, a partially edentulous maxillary and a fully dentulous mandibular typodont (Kavo basic study model, Kavo Dental GmbH, Biberbach, Germany) were utilized to create two representative reference models. Reference models were created using a polyurethane material (Alpha‐Pur, Shore A70, CTH Bezema R. Beitlich GmbH; Tübingen; Germany). One abutment tooth (a maxillary canine) was prepared to receive a veneer, one abutment tooth (a maxillary first incisor) was prepared to receive a full crown, and two abutments (a maxillary first premolar and a second molar) were prepared to receive a single‐span four‐unit FPD. A computer‐aided impression (CAI) was taken with the 3M True Definition Scanner (software version 4.0.3.1, 3 M ESPE, St. Paul, MN, USA). This intraoral scanner (IOS) requires a scanning powder; the applied high‐resolution 3M scanning powder spray (3 M ESPE, St. Paul, MN, USA) consists of titanium dioxide, amorphous silica, aluminum hydroxide, and synthetic amorphous silica (Figure 1).

**FIGURE 2:**
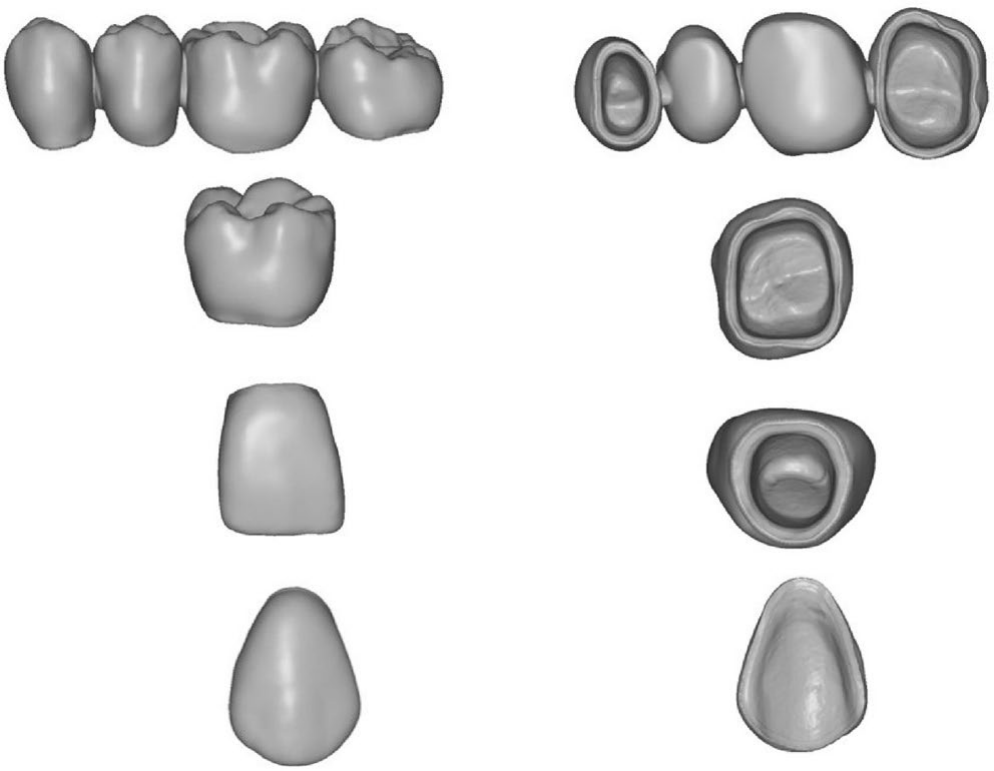
CAD design of the prospective 3D‐printed fixed dental restorations.

The size of the scanning powder particles is given as approximately 20 μm (manufacturer information). STL files were created after the scanning process and were utilized to fabricate the virtual fixed dental restorations using the CAD software (Zirkonzahn. Modellier 2015.10, Gais, Italy) (Figure 2).

**FIGURE 3:**
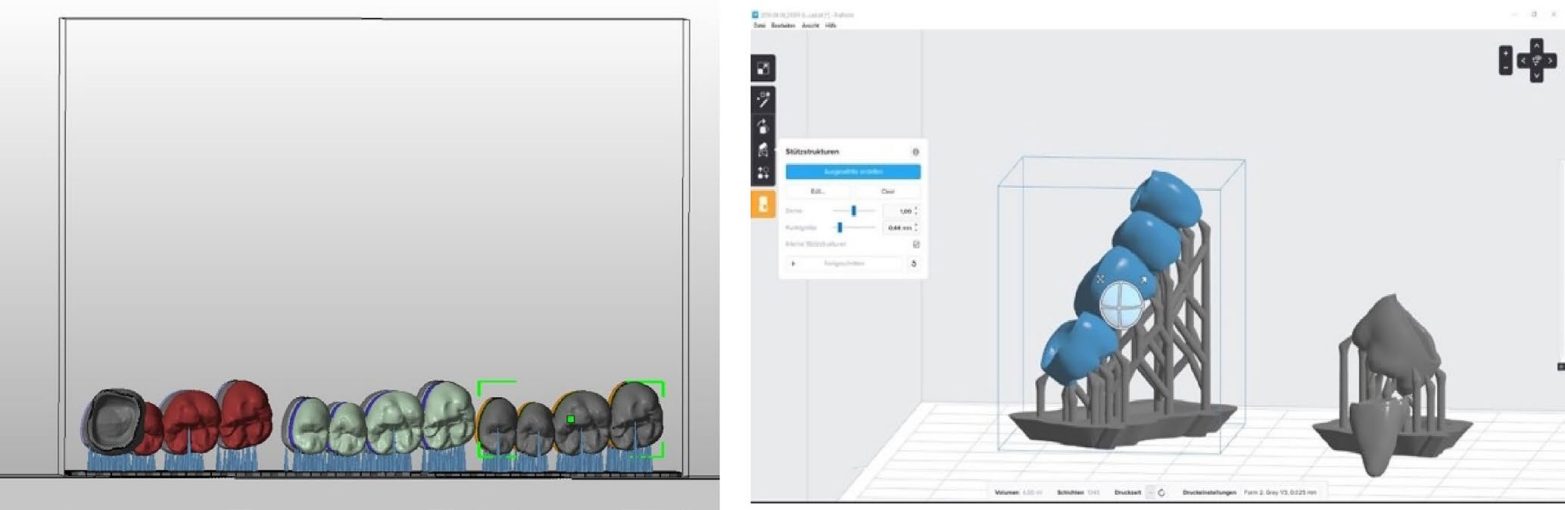
Virtual orientation of the 3D‐printed fixed dental restorations on the virtual build platform.

Two 3D‐printing technologies were utilized. For the DLP technology, the SHERAeco‐print 30 (SHERA Werkstoff‐Technologie GmbH & Co. KG, Lemförde, Germany) was utilized. Support structures were first created along the occlusal/incisal and the buccal/labial surfaces of the virtual designed restorations with a 30‐angle. The restorations were sliced into layers and sent to the 3D printer to start the printing process. SHERAprint‐cb resin in shade A3 (SHERA Werkstoff‐Technologie GmbH & Co. KG, Lemförde, Germany) was used to 3D‐print the provisional FDPs. It is a light‐curing resin that offers high detail accuracy, esthetic quality, and compatible with DLP 3D printers. The printing was performed with a layer thickness of 50 μm, following the manufacturer's instructions, using LED light with a wavelength of 385 nm. SHERAprint‐cb requires post‐curing to fully harden and stabilize the material. The printing process of restorations took one hour. For the SLA technology, the Formlabs Form 2 (Formlabs, Boston, USA) was utilized. Support structures were created along the occlusal/incisal and the buccal/labial surfaces of the virtual designed restorations with 45° angle (Figure 3). The restorations were sliced into layers and sent to the 3D printer to start the 3D printing process. Formlabs UV Gray Resin (UV Grau, Formlabs, Boston, USA) is a light‐curing resin compatible with SLA 3D printers. Is effective for prototyping fixed partial dentures, allowing for detailed and precise design and fit. The resin was used to 3D‐print FDPs with a layer thickness of 25 μm, following the manufacturer's instruction, employing an ultraviolet laser with a power of 250 mW, and the process took 8 h and 18 min. A post‐curing step is also required to fully harden and stabilize the material.

**FIGURE 4:**
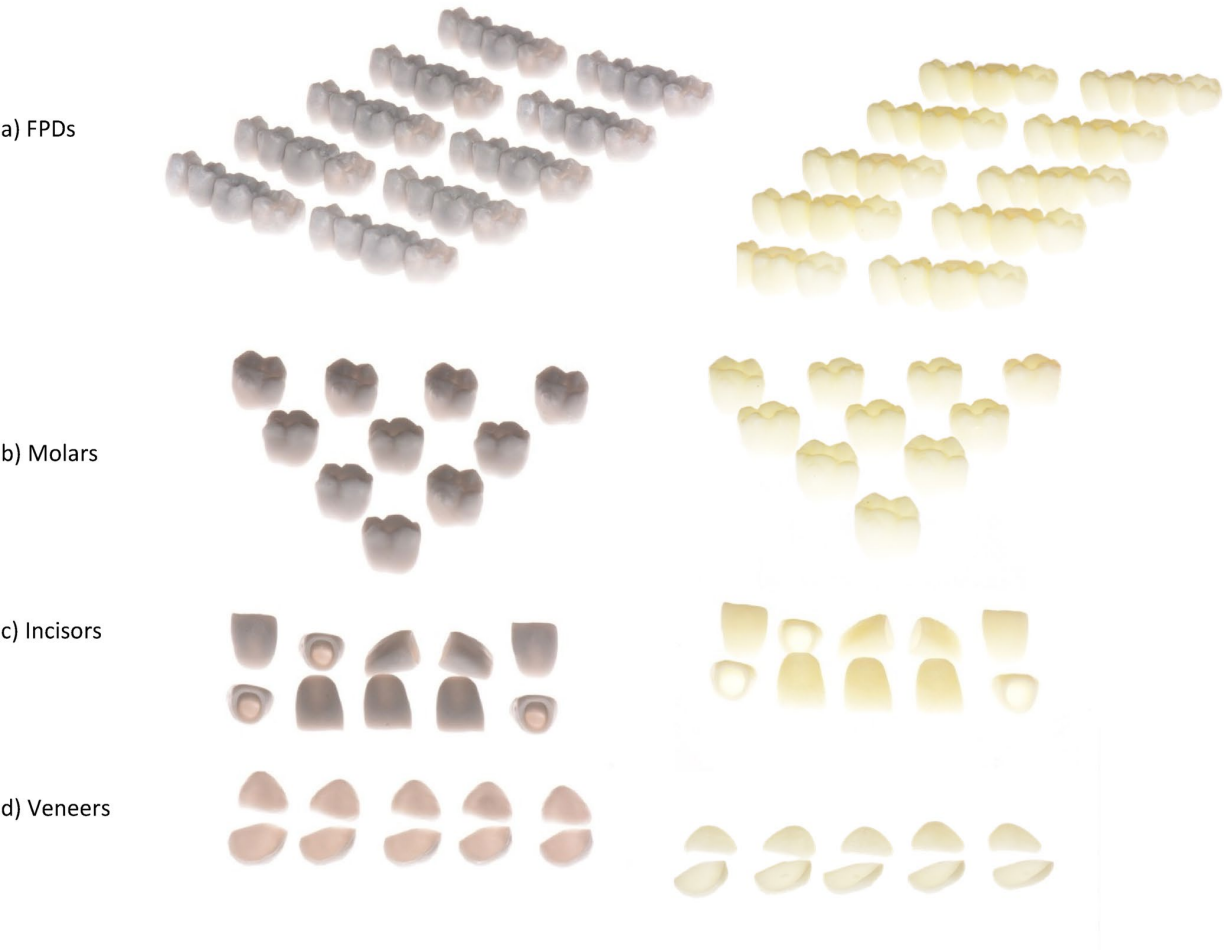
3D‐printed restorations after ultraviolet post‐curing and support structures removal (a) FPDs, (b) molars, (c) incisors, and (d) veneers.

After the 3D printing for both 3D printers was completed, all restorations were ultrasonically cleaned with 96% alcohol and post‐cured using an ultraviolet curing unit. Before performing further analyses, all restorations were visually inspected to ensure that they were free from any voids, defects, or surface roughness. The support structures were manually removed, and restorations were carefully polished (Figure 4).

### 2.2 Data Analysis

Each 3D printer technology generated 10 specimens of each type of restoration for a total of 80 restorations. The sample size of 10 per group was determined based on the error rates of the two 3D printers used, the Formlabs Form 2 (SLA) and SHERAeco‐print 30 (DLP), which have standard deviations of 10 and 15 μm, respectively [29, 32]. Using a 95% confidence level and a margin of error of 20 μm, the required sample size for individual printers was less than or equal to 2. The required sample per restoration per printer was approximately 10. These generated physical restorations were scanned again and digitized with the same IOS for evaluations. The accuracy of the 3D‐printed restorations was evaluated using the Geomagic Qualify 2012 (Geomagic, Morrisville, NC, USA). The STL files of the originally designed restorations (CAD) and the STL files of the 3D‐printed restorations were imported to the Geomagic software. The digital datasets obtained for each of the restorations were aligned and superimposed using the best‐fit algorithm method to measure the discrepancies. The best‐fit algorithm moves the test object to the reference object using an iterative closest‐point algorithm (ICP); this step was performed automatically [41]. Distributions of the discrepancies were presented as color‐coded maps with 21 color segments. The range of maximum and minimum nominal values was set at ±20 μm and the maximum and minimum critical values were set at ±200 μm. The yellow‐to‐red color illustrates that the tested restoration is larger than the reference restoration (positive values). The turquoise‐to‐blue color illustrates that the tested restoration is smaller than the reference model (negative values). To ensure consistency and minimize variability, all 3D printing, post‐processing, scanning, and evaluation procedures were conducted by a single skilled operator (A.A), following a standardized protocol across all trials. This approach aimed to enhance the repeatability of the results and reduce potential discrepancies related to operator variability, a critical factor in maintaining accuracy [42].

Two parameters were evaluated to describe the accuracy of the restorations. According to the description in ISO 5725‐1:2023, accuracy consists of trueness and precision [43]. Trueness refers to the closeness of agreement between the arithmetic mean of a large number of test results and the true or accepted value [43]. In this in vitro study, the trueness value was defined as the deviation from the originally designed CAD restorations. Precision refers to the variability between repeated measurements [43]. In this in vitro study, the precision value was defined as the variation of deviations generated among the 3D‐printed FDPs. These measurements are critical for ensuring accurate and reliable outcomes in restorations [44].

**FIGURE 5:**
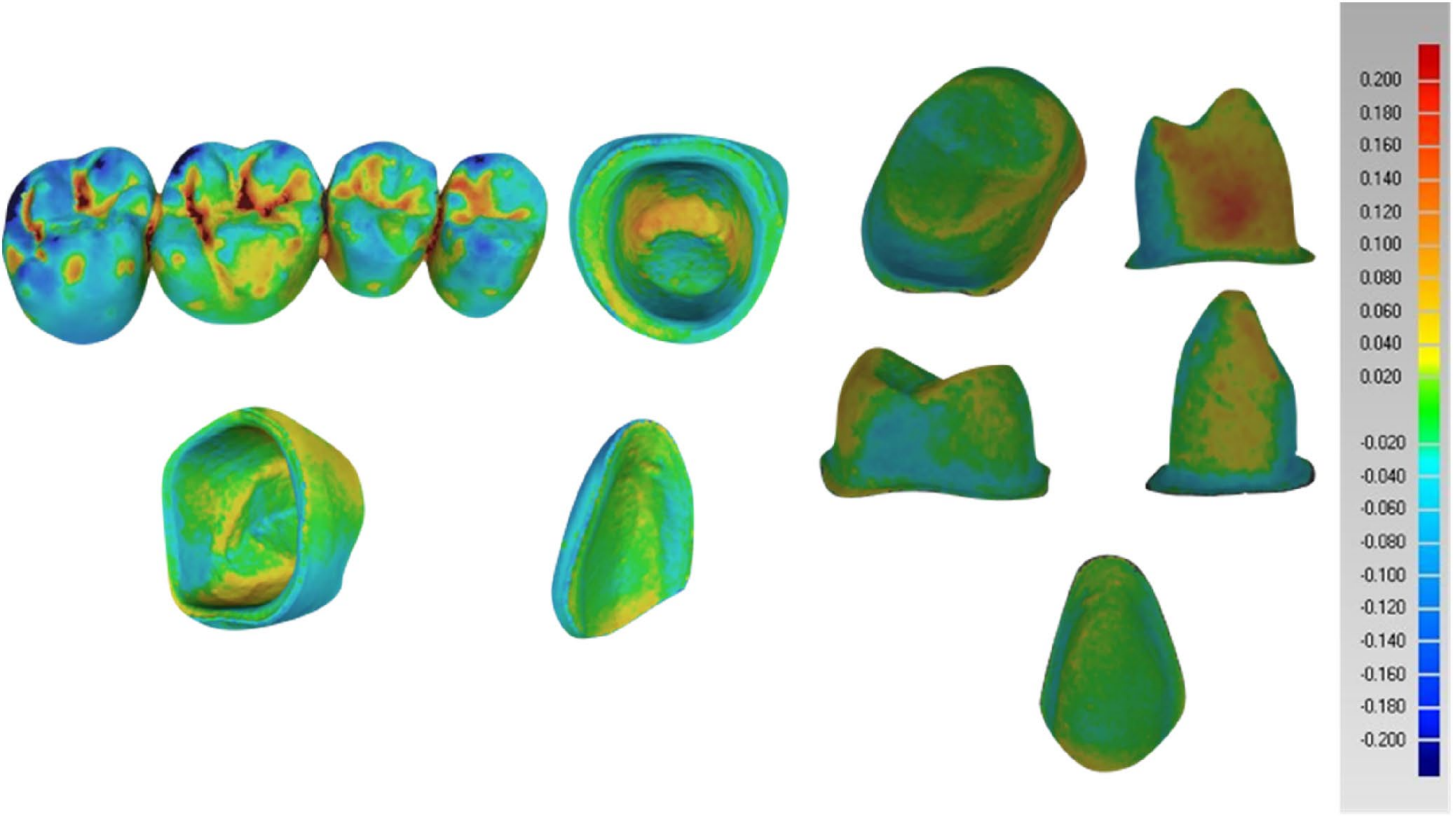
Three‐dimensional analysis of the 3D‐printed fixed dental restorations and copings using the Geomagic software (Qualify 2012).

The statistical analysis of each restoration is represented by the overall evaluation concerning the external dimensional analysis of the labial surfaces of the veneers, labial and palatal surfaces of the incisors, occlusal surfaces of the molars, and the FPDs as well as the internal dimensional analysis that is represented as the copings of the FDPs (Figure 5). The analysis was performed using the statistical software (Minitab 16 Version 16.1.1, Minitab Inc., USA). One‐way ANOVA and a two‐sample T‐test were used to test the overall trueness. A “two variances” test was performed to evaluate the differences in precision. A level of statistical significance (*p*‐value < 0.05) was set.

## 3 Results

**FIGURE 6:**
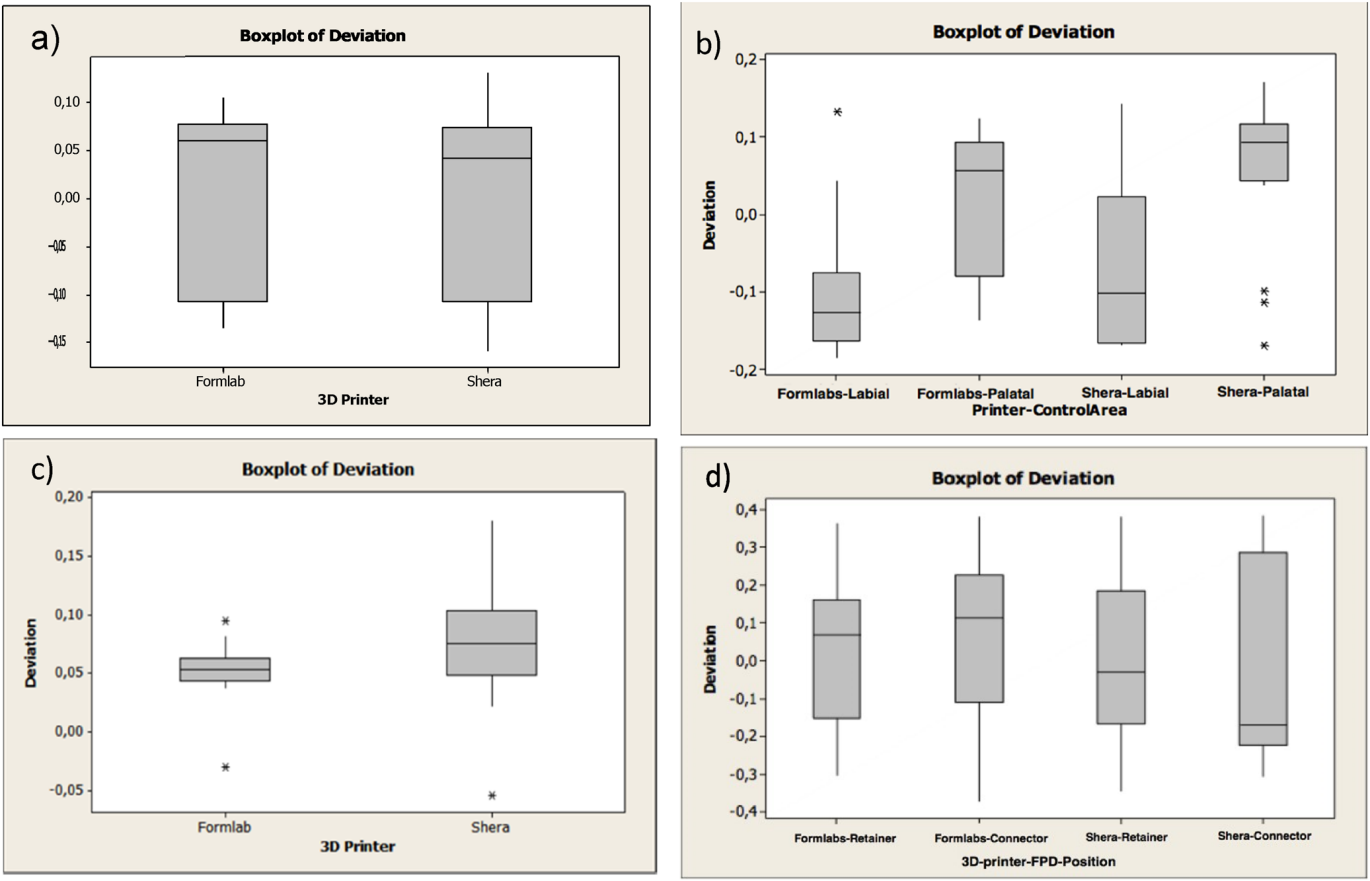
Boxplots representing the trueness of the external dimensional changes of the 3D‐printed fixed dental restorations; (a) veneers, (b) incisors, (c) molars, (d) retainers of the FPDs (in micrometers). * = outlier.

Table 1 summarizes the overall trueness values of the external and internal dimensional analysis of the 3D‐printed restorations. Positive values indicate that the 3D‐printed restorations are larger than the reference CAD design and negative values indicate that the 3D‐printed restorations are smaller than the reference CAD design. Regarding the external dimensional analysis of the incisors, it was found significant differences in the trueness (*p*‐value ≦ 0.001). On the labial surfaces, SLA 3D‐printed resin‐based incisors showed a trueness of −97 ± 84 μm and DLP 3D‐printed incisors showed a trueness value of −77 ± 98 μm (Figure 6b).

**TABLE 1:** Overall trueness values of the 3D‐printed fixed dental restorations.

Trueness			
Volumetric changes	*p*	Formlabs (SLA)	Shera (DLP)
Veneers	0.854	88 ± 26 μm	85 ± 41 μm
Incisors labial	0.001	−97 ± 84 μm	−77 ± 98 μm
Incisors palatal		22 ± 83 μm	64 ± 91 μm
Molars	0.002	53 ± 19 μm	77 ± 42 μm
FPDs^a^	0.004	181 ± 91 μm	214 ± 89 μm

Copings	*p*	Formlabs (SLA)	Shera (DLP)
Veneers	0.909	18 ± 7 μm	17 ± 2 μm
Incisors	0.012	31 ± 4 μm	52 ± 20 μm
Molars	0.001	23 ± 2 μm	31 ± 4 μm
FPDs^a^ # 14	0.001	47 ± 9 μm	52 ± 6 μm
FPDs^a^ # 17		25 ± 2 μm	44 ± 6 μm

^a^ FPDs = Fixed partial dentures.

**FIGURE 7:**
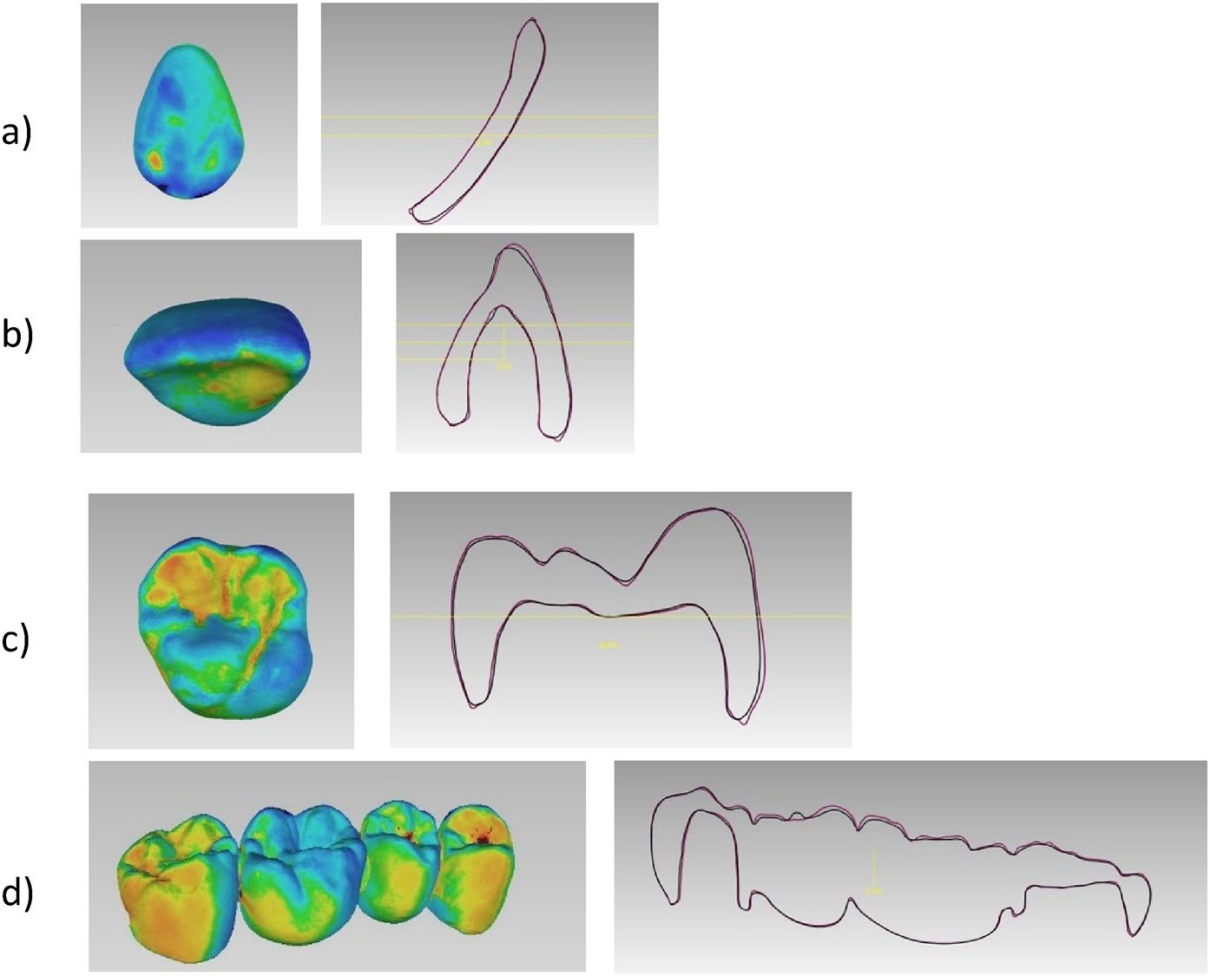
A cross section of the superimposed CAD fixed dental restorations (purple lines) and the 3D‐printed fixed dental restorations (black lines) demonstrated for the DLP; (a) veneers, (b) incisor, (c) molar, and, (d) four‐unit FPD.

**FIGURE 8:**
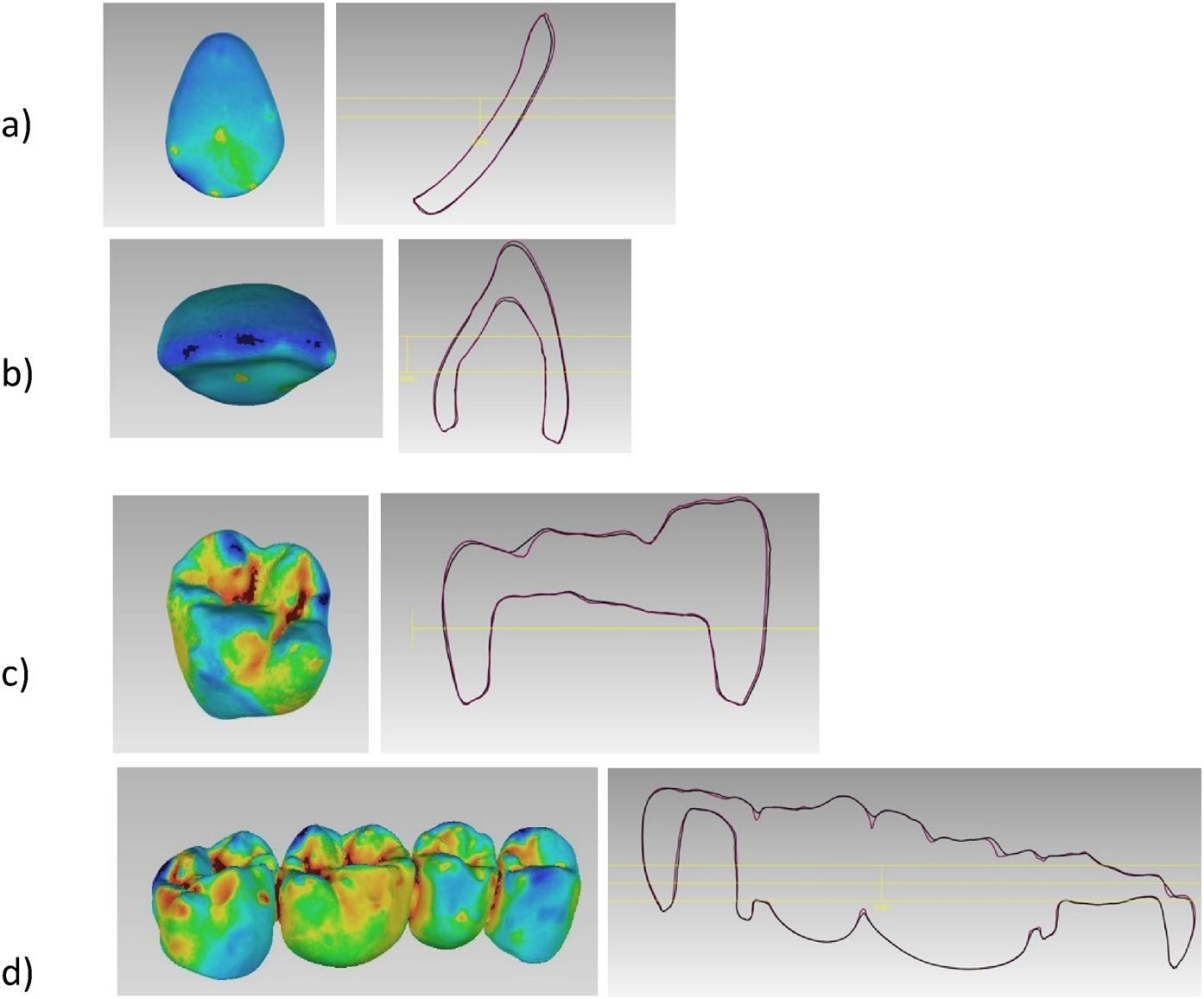
A cross section of the superimposed CAD fixed dental restorations (purple lines) and the 3D‐printed fixed dental restorations (black lines) demonstrated for the SLA; (a) veneers, (b) incisor, (c) molar, and, (d) four‐unit FPD.

Furthermore, significant differences in the trueness of the occlusal status of molars were found (*p*‐value = 0.002). DLP 3D‐printed resin‐based molars showed high trueness values of 77 ± 42 μm. Also in the FPDs, significant statistical differences of the occlusal status were found in the trueness (*p*‐value = 0.004). The DLP 3D‐printed resin‐based showed high trueness values of 214 ± 89 μm. The trueness in veneers did not show significant differences (*p*‐value = 0.854) (Figure 6c,d). Additionally, cross‐sectional superimpositions of the CAD restorations were performed for both SLA and DLP 3D‐printed restorations (Figures 7 and 8). These illustrated the trueness of the external dimensions of the restorations.

**FIGURE 9:**
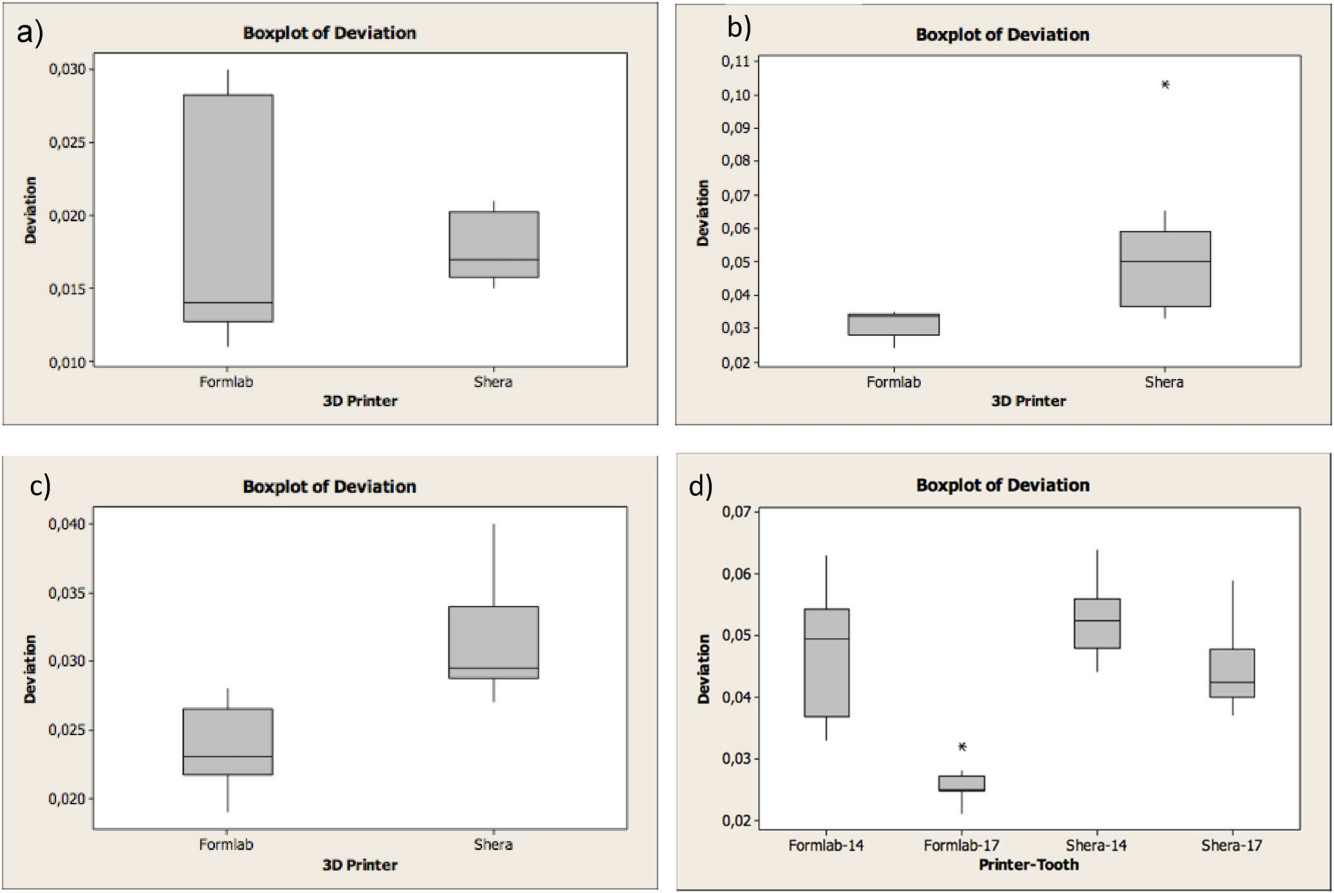
Boxplots representing the trueness of the internal dimensional changes (copings) of the 3D‐printed FDPs (a) veneers, (b) incisors, (c) molars, (d) retainers of the four‐unit FPDs (in micrometers). * = outlier.

**FIGURE 10:**
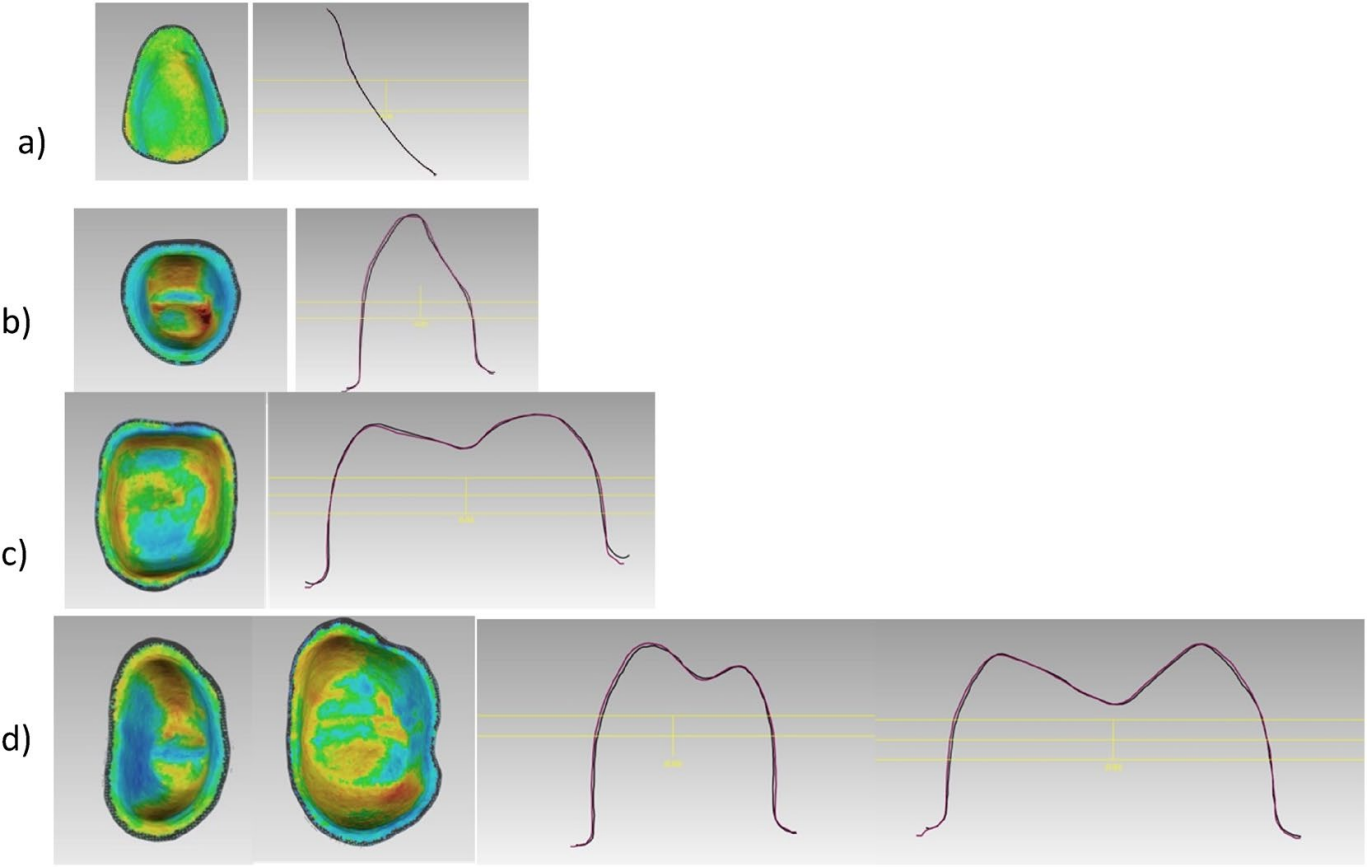
A cross section of the superimposed CAD copings (purple lines) and the 3D‐printed copings (black lines) demonstrated for the DLP; (a) veneer, (b) incisor, (c) molar, and (d) four‐unit FPD.

**FIGURE 11:**
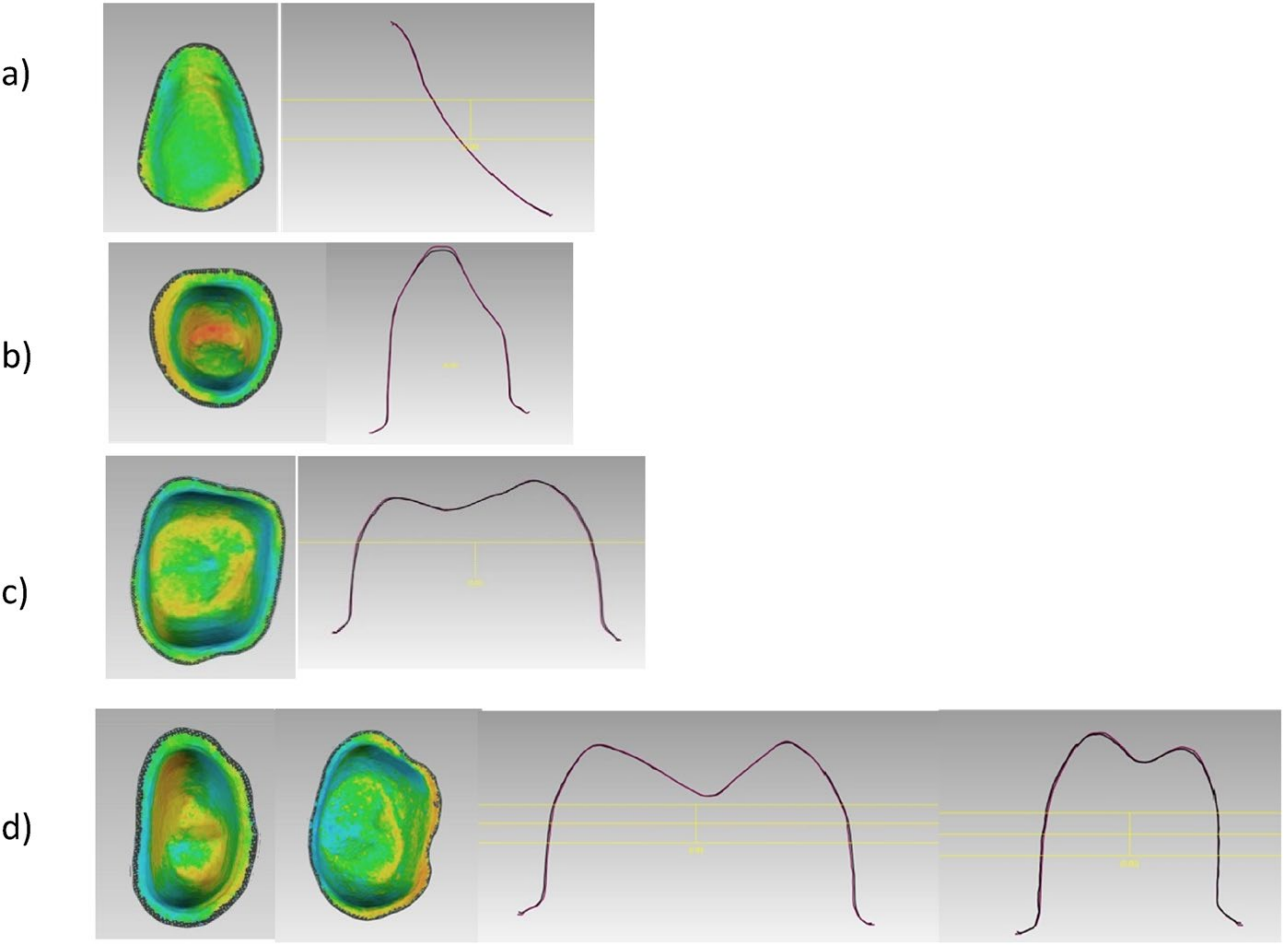
A cross section of the superimposed CAD copings (purple lines) and the 3D‐printed copings (black lines) demonstrated for the SLA; (a) veneer, (b) incisor, (c) molar, and (d) four‐unit FPD.

Copings of the incisors, molars, and four‐unit FPDs were statistically significantly different in trueness with a *p*‐value < 0.05. DLP 3D‐printed resin‐based showed high trueness values of 52 ± 20 μm. On the other hand, there were no statistical differences in the trueness values in the veneers with a *p*‐value = 0.909 (Figure 9a–d). Cross‐sectional superimpositions of the CAD copings were performed for both SLA and DLP 3D‐printed copings (Figures 10 and 11). These illustrated the trueness of the internal dimensional analysis.

**FIGURE 12:**
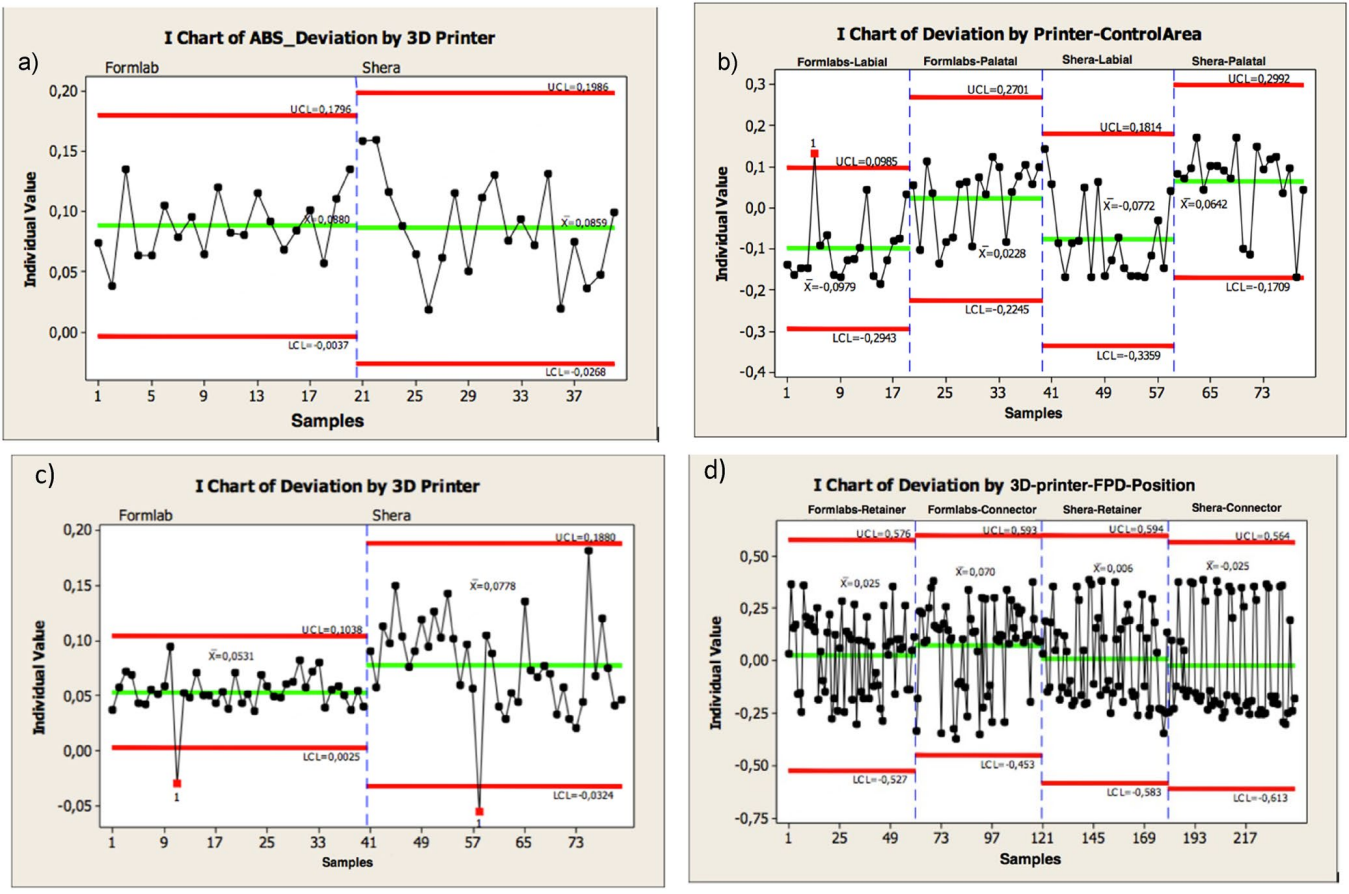
Individual chart showing the precision of the external dimensional changes of the 3D‐printed fixed dental restorations; (a) veneers, (b) incisors, (c) molars, (d) four‐unit FPDs (in micrometers). *LCL = Lower control limit; UCL: Upper control limit.

**FIGURE 13:**
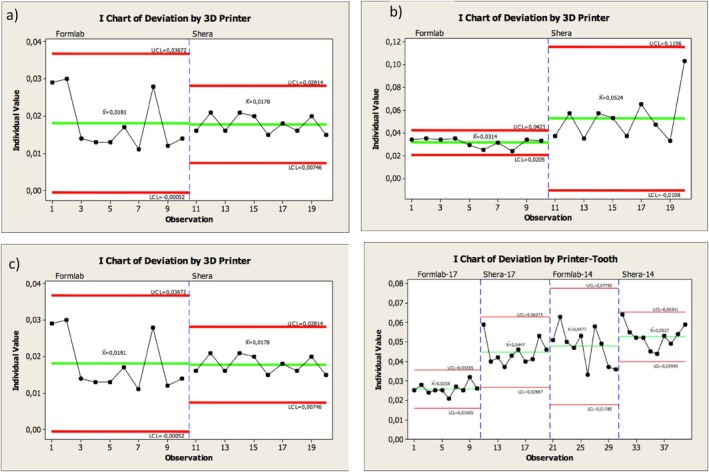
Individual chart showing the precision of the internal dimensional changes (copings) of the 3D‐printed fixed dental restorations; (a) veneers, (b) incisors, (c) molars, (d) four‐unit FPDs (in micrometers).

Table 2 summarizes the overall precision values of the external and internal dimensional analysis of the 3D‐printed restorations. Furthermore, Figure 12a–d illustrates the control limits for the external dimensional changes of the 3D‐printed fixed dental restorations, highlighting the variability among the restorations. Marginal statistical significance in the precision of the external dimensional analysis of the veneers was found (*p*‐value = 0.054). Statistically significant differences in the precision of the molars occlusally were found (*p*‐value ≦ 0.001). High variability between the 3D‐printed molars of the DLP 3D‐printed ranging between −32 and 188 μm (lower and upper control limits) was found (Figure 12c). Figure 13a–d illustrates the control limits for the internal dimensional changes of the 3D‐printed fixed dental restorations, highlighting the variability among the restorations. Copings of the veneers, incisors, and retainers of the FPDs showed statistically significant differences in the precision *p*‐value = 0.002, ≦ 0.001, and 0.012, respectively. Copings of the SLA 3D‐printed resin‐based incisors showed higher variability ranging between 0.5 and 36 μm (lower and upper control limits), while copings of the DLP 3D‐printed resin‐based incisors showed higher variability ranging between 1 and 11 μm (lower and upper control limits) (Figure 13b). No statistical differences in the precision of the molars were found (*p*‐value = 0.305) (Figure 13c).

**TABLE 2:** Overall precision values of the 3D‐printed fixed dental restorations.

Precision			
External dimensional changes	*p*	Formlabs (LCL–UCL)^a^ (SLA)	Shera (LCL‐UCL)^a^ (DLP)
Veneers	0.054	−3–179 μm	−26–198 μm
Incisors labial	0.892	59–139 μm	69–160 μm
Incisors palatal		59–137 μm	64–149 μm
Molars	≦ 0.001	2–103 μm	−32–188 μm
FPDs^b^	0.101	169–270 μm	206–328 μm

Internal dimensional changes	*p*	Formlabs (LCL–UCL)^a^ (SLA)	Shera (LCL‐UCL)^a^ (DLP)
Veneers	0.002	0.5–36 μm	7–28 μm
Incisors	≦ 0.001	2–4 μm	1–11 μm
Molars	0.305	1–5 μm	2–8 μm
FPDs^b^ # 14	0.012	6–12 μm	3–13 μm
FPDs^b^ # 17		1–6 μm	4–14 μm

^a^ LCL = Lower control limit; UCL: Upper control limit.

^b^ FPDs = Fixed partial dentures.

## 4 Discussion

3D‐printed dental restorations are created using a layer‐by‐layer fabrication method. This in vitro study examined the accuracy of different resin‐based 3D‐printed fixed dental restorations. The working hypothesis was partially rejected, as the restorations produced by the 3D printers did not achieve the ideal clinical level of accuracy. Despite this, the number of studies exploring the accuracy of 3D‐printed fixed dental restorations is growing [33, 39, 45, 46, 47]. The aim of utilizing this technology is to establish an alternative, reliable fabrication method that can either replace or complement subtractive and traditional fabrication techniques.

Digitizing or data acquisition of reference models using IOS was the first step of the digital workflow in this investigation. The accuracy of this process directly influences the adaptation and fit of various types of dental restorations [48]. It was concluded that IOS can produce accurate FDPs in partially edentulous arches [49]. The IOS used in this present study is the True Definition Scanner. Currently, this IOS is not commercially available in the market. It is a video‐based system that captures the desired intraoral surfaces in a continuous series of images, which in turn increases the accuracy of the captured data [50, 51]. Previous and recent studies reported its accuracy for single, partial, and full arch scanning [52, 53, 54, 55, 56]. Furthermore, recent studies on the 3 M True Definition scanner have highlighted the influence of scanning powder on accuracy [52, 55, 57]. The powder is used to enhance the scanner's ability to capture precise data by improving surface reflectivity, especially on shiny or translucent surfaces [58, 59]. While it can improve scan accuracy, the powder introduces potential challenges, such as altering surface geometry slightly and requiring uniform application to avoid inconsistent data acquisition [60]. Despite these issues, correct application of the powder according to the manufacturer's instructions enhances the accuracy of the scans, making it a valuable tool in capturing detailed dental impressions [61, 62].

In this in vitro study polymer‐based materials provided from both 3D‐printer manufacturers were used. Since, using the material provided by the same 3D printer manufacturer enhances production accuracy [29, 38]. The internal dimensional analysis showed that the trueness of all types of the tested FDPs for both 3D‐printing technologies is within the ideal clinical level of acceptance ranging between 17 and 52 μm. Internal dimensional or coping analysis can also be evaluated by measuring the marginal gap width or the absolute marginal discrepancy [63]. This in vitro study evaluated the trueness and precision of the copings of all types of 3D‐printed FDPs through the 3D software Geomagic (Qualify 2012). Here, the intaglio surfaces of the reference CAD models and the tested FDPs were marked, removed, fitted together through the best‐fit algorithm step, and then 3D compared.

This method is simple to manipulate and takes less time since each fixed dental restoration was analyzed for the external and internal dimensional changes at the same time. However, this method did not analyze the accuracy of the internal fit of the restorations on the reference physical models. The accuracy was measured virtually according to the CAD reference model. Therefore, the coping analysis values may involve some technical errors related to the best‐fit algorithm and the 3D compare steps.

Ideal marginal fit is the key criterion for the success of FPDs. Discrepancies in the marginal fit lead to adherence of oral bacteria, which contributes to plaque accumulation, secondary carries, cement microleakage, and endodontic inflammations [64]. A diversity of values and tested methodologies were published in the literature to access the maximum clinically accepted values. These values ranged between 50 and 150 μm [65, 66, 67]. In a systematic review, it was found that 3D‐printed FPDs fabricated from polymer‐based materials have superior marginal fit and internal adaptation and can be used as an alternative and valid fabrication method [47]. However, great variability in precision was detected between the 3D‐printed restorations, indicating that the reproducibility of both printers is negligible.

Concerning the external dimensional accuracy, the overall trueness values found that the occlusal surfaces of the four‐unit FPDs ranged between 181 ± 91 μm (SLA) and 214 ± 89 μm (DLP). Also, trueness values of the occlusal surfaces of the molars ranged between 53 ± 19 μm (SLA) and 77 ± 42 μm (DLP). Clinically, those restorations cannot be further processed and cemented without additional occlusal chairside adjustments. Some in vitro studies showed disparities in the external dimensional accuracy of 3D‐printed four‐unit FPDs, these were either larger or smaller than the CAD model [68, 69]. Furthermore, it was shown that the post‐curing step might influence the increase in dimensional accuracy [70].

Both SLA and DLP 3D‐printed restorations of the incisors showed significant differences. The observed deviations on the buccal surfaces were in negative values ranging between −97 ± 84 μm (SLA) and − 77 ± 98 μm (DLP). This is represented in the color‐coded scales by the turquoise to dark blue areas, which indicated that the printed restorations were smaller than the reference model or in other words a shrinkage of the printed parts occurred. However, these deviations can be attributed to the distinct two‐plane morphology of the incisors, which affects accurate fitting to the CAD model [71]. The best‐fit algorithm procedure's technical sensitivity further contributes to these discrepancies, resulting in higher deviations [72].

It was proven that SLA technology provides better dimensional accuracy over other 3D‐printing technologies [73], which was also observed in this in vitro study. This might be due to the longer printing time that provides adequate polymerization of the 3D‐printed parts [74]. However, other parameters might influence the accuracy of 3D printing. Build orientation angle and layer thickness are the most studied and influential parameters reviewed in the literature [29, 74, 75, 76, 77]. Build angle is the orientation of the printed model concerning the printer build platform. As the build angle changes, the geometry of the printed layers changes. This, in turn, changes the supporting relationship of a given print layer to its successively printed layers, affecting ultimately the print accuracy in that region [76, 78, 79]. Studies have shown that 30° and 45° build angle orientations provide acceptable dimensional accuracy and surface finish [75, 76, 77, 80]. Moreover, a build angle within these ranges optimizes the model's self‐supporting capabilities, minimizing the necessary support surface area [29]. It does not only enhance the model's dimensional accuracy but also simplifies the manual removal of support structures without significantly impacting the dimensional accuracy of the 3D‐printed restorations [29, 81]. Build orientation angles in this in vitro study were chosen according to the literature reviews and 3D‐printer manufacturers' recommendations [73, 82, 83].

Layer thickness represents the height of each layer of the 3D‐printed material regardless of the technology utilized. Layers are built along the Z coordinate and known as Z‐axis resolution [79]. In general, the effect of layer thickness on the print accuracy depends on the 3D printing technology and the geometrical complexity of the dental restoration [84]. Controversial results were found in the literature regarding the effect of layer thickness on accuracy. Studies recommended setting high layer thickness in the range of 100 μm [85, 86, 87], while others found that lower layer thickness ranging between 25 and 50 μm provided more accurate dental restorations [82, 84, 88, 89]. It was also found that the quality of 3D‐printed FDPs with the 100 μm layer thickness was similar to that of the 50 μm layer thickness [75].

In vitro studies do not always mimic clinical conditions and the application of AM technologies remains still limited. Several challenges facing SLA and DLP technologies remain to be addressed [90]. Polymer‐based 3D‐printing primarily depends on photopolymer materials [19]. This restricts the range of resins that can be utilized, presenting significant disadvantages when compared to subtractive and traditional fabricating techniques [91]. Furthermore, support structure material cannot be recycled [92], and post‐processing procedures are required, which adds time and effort to the overall production workflow [93]. Future materials will increasingly focus on producing objects that eliminate the need for post‐processing step [94]. A notable example is the capability to print ceramic‐like materials for final restorations [91]. Recent advancements are also increasingly centered on the integration of artificial intelligence (AI) with 3D‐printing technologies, aiming to facilitate the rapid production of complex dental prosthesis with enhanced accuracy [76]. By leveraging AI, the development of 3D‐printing technology is accelerating in both software—such as in designing and nesting—and hardware manufacturing processes [94], AI can analyze tooth anatomy in detail and recommend appropriate materials, leading to the creation of accurate crowns, bridges, and dentures. This integration promises to deliver faster, more cost‐effective, and clinically acceptable solutions for patients [95].

## 5 Conclusion

Within the limitations of this current in vitro study, the following conclusions are drawn:

- The internal dimensional accuracy of both SLA and DLP polymer‐based fixed dental restorations was within clinically acceptable ranges (17–52 μm).
- The external dimensional accuracy was less optimal across both technologies and materials used.
- The 3D‐printed fixed dental restorations can be clinically acceptable with chairside modifications.
- The significant statistical differences and high variability observed suggest that the reproducibility of both 3D printers was not optimal.

## Conflicts of Interest

The authors declare no conflicts of interest.

## Data Availability

Research data not shared.
